# Task-uninformative visual stimuli improve auditory spatial discrimination in humans but not the ideal observer

**DOI:** 10.1371/journal.pone.0215417

**Published:** 2019-09-09

**Authors:** Madeline S. Cappelloni, Sabyasachi Shivkumar, Ralf M. Haefner, Ross K. Maddox

**Affiliations:** 1 Biomedical Engineering, University of Rochester, Rochester, New York, United States of America; 2 Del Monte Institute for Neuroscience, University of Rochester, Rochester, New York, United States of America; 3 Brain and Cognitive Sciences, University of Rochester, Rochester, New York, United States of America; 4 Center for Visual Science, University of Rochester, Rochester, New York, United States of America; 5 Neuroscience, University of Rochester, Rochester, New York, United States of America; Harvard Medical School, UNITED STATES

## Abstract

In order to survive and function in the world, we must understand the content of our environment. This requires us to gather and parse complex, sometimes conflicting, information. Yet, the brain is capable of translating sensory stimuli from disparate modalities into a cohesive and accurate percept with little conscious effort. Previous studies of multisensory integration have suggested that the brain’s integration of cues is well-approximated by an ideal observer implementing Bayesian causal inference. However, behavioral data from tasks that include only one stimulus in each modality fail to capture what is in nature a complex process. Here we employed an auditory spatial discrimination task in which listeners were asked to determine on which side they heard one of two concurrently presented sounds. We compared two visual conditions in which task-uninformative shapes were presented in the center of the screen, or spatially aligned with the auditory stimuli. We found that performance on the auditory task improved when the visual stimuli were spatially aligned with the auditory stimuli—even though the shapes provided no information about which side the auditory target was on. We also demonstrate that a model of a Bayesian ideal observer performing causal inference cannot explain this improvement, demonstrating that humans deviate systematically from the ideal observer model.

## Introduction

As we navigate the world, we gather sensory information about our surroundings from multiple sensory modalities. Information gathered from a single modality may be ambiguous or otherwise limited, but by integrating information across modalities, we form a better estimate of what is happening around us. While our integration of multisensory information seems effortless, the challenge to the brain is non-trivial. The brain must attempt to determine whether incoming information originates from the same source, as well as estimate the reliability of each modality’s cues so that they may be appropriately weighted.

Studies of multisensory integration have explained how a Bayesian ideal observer could solve this problem by combining reliability-weighted evidence from multiple sensory modalities. In the forced integration model, an observer gathers evidence from multiple modalities and combines them according to the modality’s reliability [1]. Importantly this allows for the most reliable sensory estimate to dominate the percept while noisier measurements have less influence; however, it also implies that percepts of distinct stimuli that in actuality originate from independent sources must nonetheless be perceptually influenced by each other. More recently, causal inference has expanded upon the forced integration model by allowing the observer to treat stimuli as originating from different sources. The observer first determines whether both pieces of evidence are likely to come from a common source, and if so weights them by their reliabilities as in the forced integration model to generate a combined percept [2]. In their basic forms neither model attempts to contend with scenes more complex than a single stimulus in each modality.

Numerous experiments have shown that humans behave as ideal or near-ideal Bayesian observers performing forced integration [3–6] or causal inference [7–10]. There have even been efforts to reveal which brain structures contribute to Bayesian computations [11, 12]. However, studies rarely considered scenarios in which many sources in an environment give rise to multiple cues within each modality. Though additional auditory and/or visual stimuli in behavioral tasks have been employed to test audio-visual binding [13, 14], to increase perceptual load [15], etc., there has been no effort to use such tasks to test the limits of Bayesian models. Here we test the Bayesian causal inference model using a new paradigm, and in doing so introduce a key question missing from these prior studies, but common in the natural world: which auditory and visual stimuli will be integrated when multiple stimuli exist in each modality?

In the case of a single stimulus in each modality, visual influence on auditory location has been largely demonstrated by studies of perceptual illusions. Notably, the ventriloquist effect, a bias of auditory location toward visual location when cues of both modalities are presented simultaneously [16], has been extensively characterized. The influence of the visual location depends mainly on two factors: the discrepancy between the two stimuli (with visual-induced bias waning as the spatial separation becomes too large) [17], and the size of the visual stimulus (smaller, more reliable, visual stimuli yielding a larger bias) [4]. Dependence on discrepancy points to a causal inference structure, while size dependence indicates a weighting by the quality of the location estimates (larger visual stimuli are localized less accurately). Agreement with the Bayesian causal inference model [2, 4] would indicate that the bias is due to an integration of the two cues in which the brain produces a combined estimate of location. Therefore, congruent auditory and visual evidence should result in a more accurate estimate of object location than auditory evidence alone.

Furthermore, we explore the influence of sensory stimuli in a scene that are not related to the observer’s task. Ideal Bayesian causal inference describes a statistical inference of the correct choice based on relevant sensory information. By definition, such a model is unaffected by task-uninformative stimuli. Nonetheless, studies have shown that human behavior can be influenced by task-uninformative stimuli [18–20]. We demonstrate how these effects are not described by established models and propose a variety of alterations that may offer a more complete description of human perception.

In this study we engaged listeners in a concurrent auditory spatial discrimination task to look for a benefit from spatially aligned, task-uninformative visual stimuli. *Task-uninformative* here refers specifically to stimuli that do not provide information about the correct choice on a given trial, though they may provide knowledge about the broader sensory scene. Given only task-uninformative cues, the observer could only perform at chance. We presented two sounds, a tone and noise, with centrally located or spatially aligned visual stimuli of per-trial random color and shape. Listeners were asked to report which side the tone was on. Importantly, those shapes do not provide information about the correct choice in either condition, but do indicate the separation of the two auditory stimuli in the spatially aligned condition. We investigated whether subjects nonetheless benefited from this additional knowledge and improved their performance on the task as one might predict from an extrapolation of the ventriloquist effect. Our results show a benefit due to the spatially aligned task-uninformative shapes. However, an extension of the ideal Bayesian causal inference model for two auditory and two visual stimuli could not explain any difference in auditory performance between the two visual conditions. This difference between observed and predicted behavior suggests neural processing that goes beyond (or falls short of) the ideal observer.

## Results

### Psychophysics

**Fig 1:**
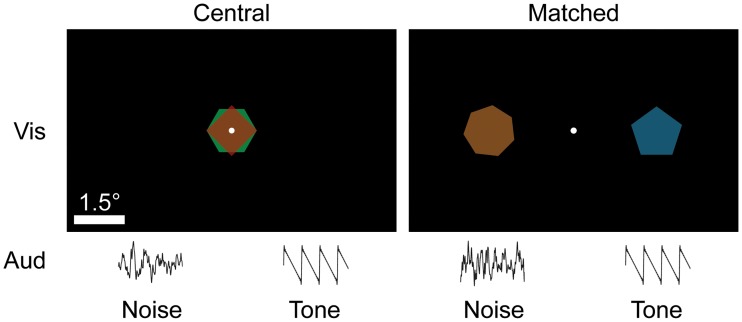
Listeners fixate while concurrently hearing two auditory stimuli on either side of the fixation dot and seeing two random shapes that are either centrally located or spatially aligned with the auditory stimuli. Shapes are presented in alternating frames to avoid overlap.

**Fig 2:**
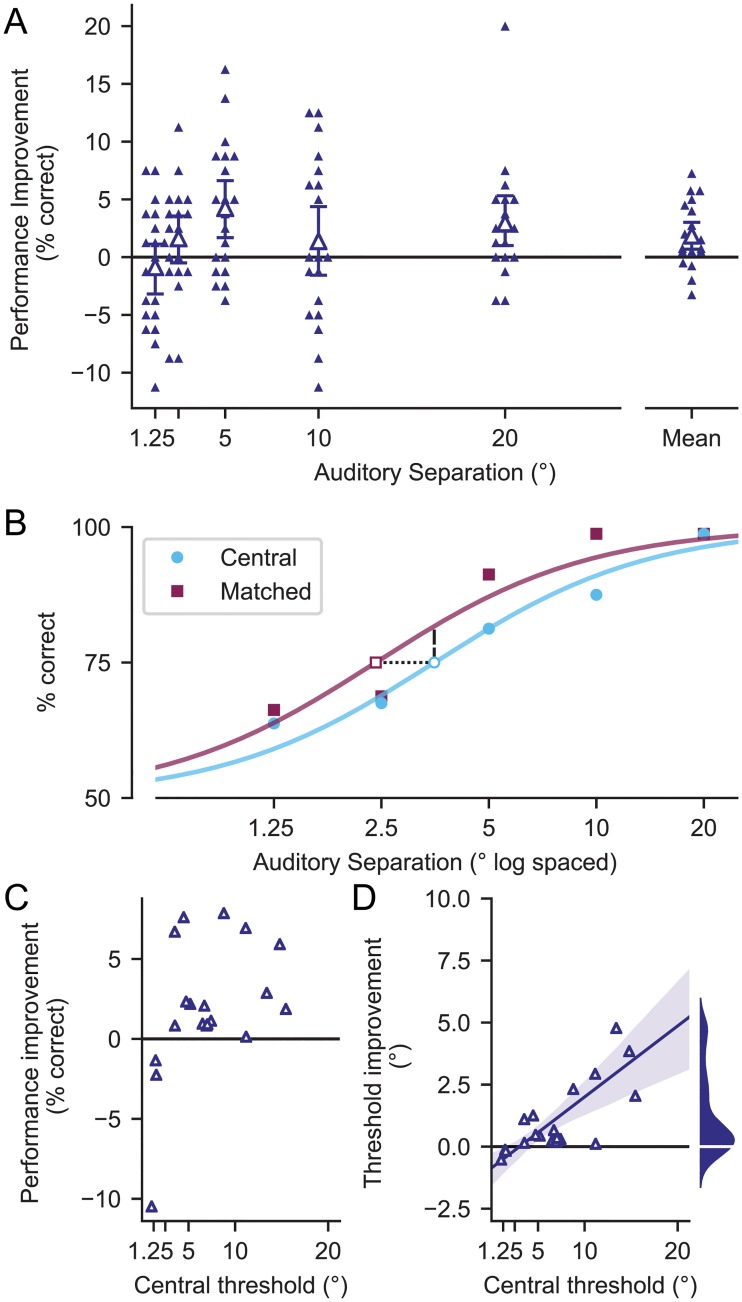
Behavioral results comparing central and matched conditions. **A)** Improvement in performance at each angle averaged across subjects. Error bars show 95% confidence intervals and individual subjects shown as small triangles. **B)**. Sigmoidal fits of the data in log units for a single subject who shows the effect. **C)**. Improvement in performance (% correct) at each subject’s separation threshold in the central condition (dashed line in B). **D)** Improvement in separation threshold (degrees) for each subject (dotted line in B). Line of best fit and 95% confidence intervals also shown. Marginal distribution of threshold improvement shown to the right. There is more mass towards positive threshold improvement than negative.

We engaged listeners in an auditory spatial discrimination task to see if they could benefit from spatially aligned task-uninformative visual stimuli. Listeners were presented with two simultaneous sounds (a tone complex and noise token with the same spectral shape) localized symmetrically about zero degrees azimuth and asked to report which side the tone was on. Concurrently, two task-uninformative visual stimuli of per-trial random shape and hue were presented. In two conditions (Fig 1) with interleaved trials, visual stimuli were either spatially aligned with the auditory stimuli (“Matched” condition) or in the center of the screen (“Central” condition) as a control. For both conditions, auditory separations ranged from 1.25 degrees to 20 degrees. We measured the improvement in performance due to the spatially aligned shapes as the difference in percent correct between matched and central conditions for each separation (Fig 2A). Averaging across separations for each subject, the 1.86% improvement was significant with (*t*(19) = 3.02, *p* = 0.007, *t*-test). The effect was individually significant at moderate and large separations (4.25% increase at 5 degrees (*t*(19) = 3.37, *p* = 0.003) and 2.94% increase at 20 degrees (*t*(19) = 2.59, *p* = 0.02)). Effect sizes across subjects and separations are highly variable due to differences in auditory spatial processing ability. At a large separation relative to the subject’s ability, performance may be at the lapse rate even in the central condition and no benefit of the visual stimulus may be observed. Conversely, at relatively small separations, any visual benefit may be insufficient to produce better than chance performance (i.e. subjects are guessing in both conditions) and we will not observe a behavioral benefit.

To further understand the effect, we calculated 75% thresholds for each condition by fitting psychometric functions to each subject’s response data (Fig 2B). Improvements in threshold across conditions and improvements in performance at threshold are shown in Fig 2C and 2D. A decrease in separation thresholds (dotted line Fig 2B) is necessarily paired with an increase in percent correct at threshold (dashed line Fig 2B) due to the fit method (slope and lapse rate of the sigmoid were determined from responses to both conditions and only the threshold parameter of the function was allowed to differ between the two conditions). Nonetheless, we find that improvements at the central separation threshold (and consequently, performance at threshold) are significant across the population (*p* = 0.0002, sign test). The average threshold improvement across the population is a 1.1 degree decrease, and the size of the effect increases as baseline auditory spatial ability gets worse. On average, someone with a 5 degree central separation threshold experiences a 0.5 degree (10%) improvement in threshold but someone with a 15 degree central threshold experiences a 3 degree (20%) improvement. The average change in performance at the central threshold is a 2.2% improvement in percent correct.

### Modeling

We developed an ideal observer model for our task in order to investigate whether our data are compatible with an optimal combination of auditory and visual cues in this task. Our model (details in Methods) follows Körding et al. [2] in performing inference over whether two cues are the result of the same event, or due to different events (“causal inference”). Cues stemming from the same event are combined according to their relative reliabilities in an optimal manner. This results in a posterior belief about the location of the auditory tone. If this posterior has more mass left of the midline, the ideal observer responds “left”, otherwise “right”.

While the ideal observer performance follows an approximately sigmoidal shape as a function of auditory azimuth as expected, the two model fits corresponding to the matched and central conditions are identical at every angle. The ideal observer’s performance is thus unaffected by the presence of the visual cues and cannot explain the empirically observed behavioral difference between the two conditions.

**Fig 3:**
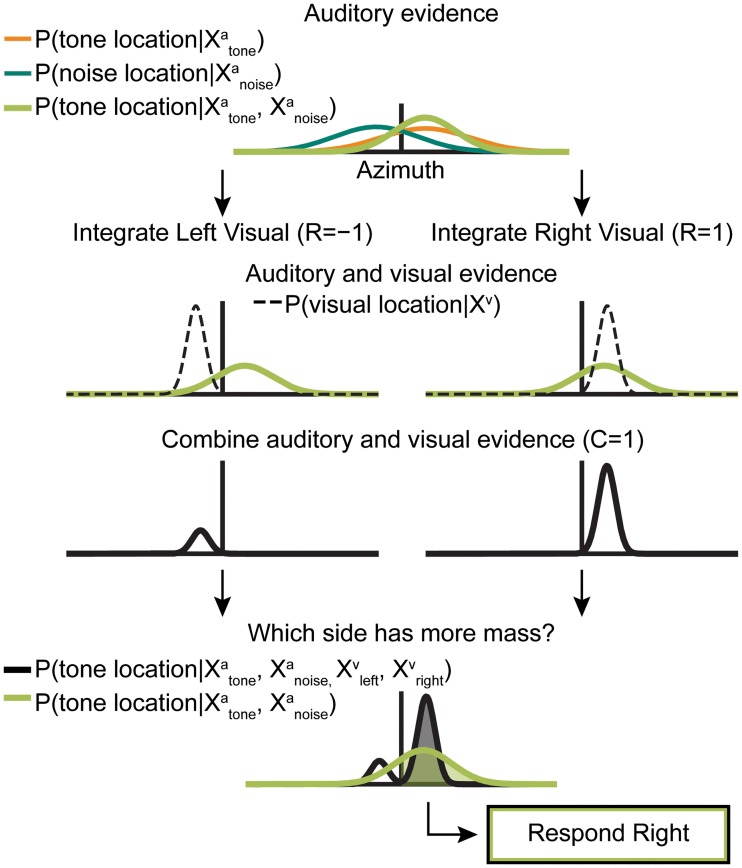
Schematic showing that visual combination cannot provide a benefit to the ideal observer. Listeners use the knowledge that the tone and noise are symmetrically presented to compute a combined auditory likelihood. Then, for each side, they combine this auditory likelihood with a visual likelihood similarly devised from both visual shapes’ likelihoods. Listeners determine which side the tone is on by picking the side of the posterior with more probability mass. Whether they do or do not combine evidence across modalities, the observer responds right.

While a full Bayesian derivation proving that the visual stimuli do not provide a benefit to the ideal observer is given in the Methods, we illustrate a simplified explanation in Fig 3. The subject’s observations imply “initial” subjective beliefs about all four stimulus locations: tone [$P(S_{\text{tone}}^{a}|X_{\text{tone}}^{a})$], noise [$P(S_{\text{noise}}^{a}|X_{\text{noise}}^{a})$], left shape [$P(S_{\text{left}}^{v}|X_{\text{left}}^{v})$], and right shape [$P(S_{\text{right}}^{v}|X_{\text{right}}^{v})$]. If the brain infers that the auditory and visual stimuli originate from a common source, all four initial beliefs are combined optimally to infer the correct task response (Fig 3). Having learned through task experience that auditory and visual stimuli are always presented symmetrically, the observer can compute a within-modality combined belief, weighting each cue by relative reliability as in Ernst & Banks [1] [$P(S_{\text{tone}}^{a}|X_{\text{tone}}^{a},X_{\text{noise}}^{a})$ and $P(S_{\text{tone}}^{a}|X_{\text{left}}^{v},X_{\text{right}}^{v})$ respectively]. Importantly, when combining with the bimodal visual likelihood, the observer must separately consider two possible scenarios: the tone is on the right, or the tone is on the left. Using the visual observation to refine their estimate of the tone location, the observer combines auditory and visual information for each scenario and must base their final decision on a weighted combination of these multisensory beliefs [$P(S_{\text{tone}}^{a}|X_{\text{tone}}^{a},X_{\text{noise}}^{a},X_{\text{left}}^{v},X_{\text{right}}^{v})$]. Even weighting the two scenarios equally, there is more evidence in favor of the tone being on the right, the same side as that implied by just the auditory observations. In reality, the weights will depend on the proximity of auditory and visual observations, favoring the visual cue that falls on the same side of the midline as the subject’s belief about the tone and will therefore yield an identical response to the one got by considering just the auditory observations. Equivalently, the side with the greater mass for [$P(S_{\text{tone}}^{a}|X_{\text{tone}}^{a},X_{\text{noise}}^{a},X_{\text{left}}^{v},X_{\text{right}}^{v})$] is the same as that for [$P(S_{\text{tone}}^{a}|X_{\text{tone}}^{a},X_{\text{noise}}^{a})$]. As a result, using the visual stimuli to refine the final posterior does not change the side with more probability mass (Fig 3), and therefore cannot benefit the ideal observer.

## Discussion

Here we show that normal hearing listeners improve their performance in an auditory spatial discrimination task when spatially aligned but task-uninformative visual stimuli are present. We further show that these findings cannot be explained by an ideal observer performing the discrimination task.

Even though the shapes presented on any given trial give no indication of which side the tone is on, subjects’ behavioral performance suggests the spatial information they provide somehow reduces errors. Since the ideal observer models must base their output only on sensory information that is informative to the correct choice, they cannot capture the difference in behavior afforded by task-uninformative stimuli, even when these stimuli provide information about the broader sensory scene. Phenomena not encapsulated by the ideal model are needed to explain these results. Assuming that the listener uses the information that the tone and noise are presented symmetrically and bases their decision on the relative positions of the two stimuli, response errors can arise from one of two situations: both auditory stimuli are perceived at the same location (respond at chance), or the relative position of the two auditory stimuli is reversed (response will be incorrect). If the listener only bases their decision on the sign of the position of the tone, errors will occur whenever the tone location estimate crosses the midline. In either scenario, we posit that visual stimuli can act as anchors to attract auditory location. The brain may therefore correct errors in auditory spatial discrimination by refining one or both auditory locations as long as it is able to correctly determine which auditory and visual stimuli to pair. Additional work must be done in order to understand how the brain accomplishes multisensory pairing.

Another interpretation of the visual benefit would be that the visual shapes help direct auditory spatial attention. The time required to shift auditory spatial attention, however, is on the order of 300 ms [21], making it unlikely that attention is driving the present results. Visual stimuli preceded the auditory stimuli by 100 ms and the auditory stimuli were only 300 ms long, a duration insufficient for the brain to redirect attention to either of the visual locations, let alone both (splitting auditory attention can have profound behavioral costs [22, 23]).

For subjects who had excellent auditory separation thresholds in the central condition, we did not observe a benefit due to spatially aligned visual stimuli. Though it is not certain whether the subjects show a true performance decrement in the matched case or simply the absence of the effect, there are two possible explanations. First, these subjects’ low thresholds indicate very good auditory spatial processing and therefore have little room to benefit from visual stimuli. These subjects may even find the visual stimuli to be a distraction. Alternatively, visual shapes will overlap in trials at 1.25 and 2.5 degrees separation. This may lead to more uncertainty in visual location in the matched condition and decrease the knowledge provided.

### The roles of early and late integration

Multisensory integration occurs throughout the sensory processing hierarchy, and can be roughly divided into early and late integration. *Early integration* is the automatic combination of multisensory evidence at low level sensory processing stages (e.g., early visual information modulating activity in primary auditory cortex). Early integration processes lead to the combination of information about stimuli that are clearly aligned in space and time, with few competing stimuli in the scene [24]. Though there is no existing mathematical framework to describe early integration, the brain should only integrate stimuli that are precisely aligned to avoid integrating information from difference sources. It is thought that stimuli integrated with early processes can capture object-based attention [25]. *Late integration* is the combination of sensory information to drive perceptual decisions, occurring at higher order processing stages. It is thought to be engaged during situations of high stimulus competition or stimulus mismatch in which top-down attention is needed to parse the scene [24]. Though there is neurophysiological and behavioral evidence of both early and late integration (see [26] for review), modeling efforts have focused on the contributions of late integration. Nonetheless, modeling has the potential to be a powerful tool for disambiguating early and late integration.

We find that the ideal Bayesian causal inference model, the canonical description of late integration, cannot account for the benefit provided by spatially aligned visual stimuli in our task. In particular, for the model observer, the visual stimuli can reduce the variance of the auditory estimate but not the side on which most of the probability mass lies, and thus the decision on a given trial never changes. Because the ideal observer cannot change their behavior based on the visual stimuli, the model is insufficient to explain the benefit we measured behaviorally. This raises the question about what is happening in the brain which could explain the improvements in empirical performance. Below we provide a systematic list of potential explanations; however, a comprehensive theoretical and experimental exploration is beyond the scope of this paper.

### Alternate models

By relaxing the constraints of the ideal Bayesian model, it is possible for the observer to benefit from sensory information that is not relevant to the perceptual decision. The following mechanisms offer potential explanations of how the brain uses the spatial information about the sensory scene provided by the visual stimuli.

**Early integration** may lead to an improvement in performance when auditory cues are automatically combined with visual cues in early sensory areas. In the matched condition, the observed auditory tone ($X_{\text{tone}}^{a}$) may be pulled towards the visual cues leading to a change in performance. Since the observed auditory tone ultimately dictates the response, improving its accuracy can explain improved performance in the task. Such automatic combination could explain not only the majority of subjects who improve their performance because the visual cue is more reliably localized but also the subjects with a decrease in performance who over-weight the visual cue even though their auditory localization is better [3].

**Bottom-up attention models** may also lead to a change in performance if the visual cue improves the sensory precision in the encoding of the auditory cues. Because there is less variance in the distribution from which the observed sample is drawn, the tone is more likely to be observed on the correct side, resulting in overall improved performance.

**Model mismatch** between the subject’s model and the experimenter’s model may result in biases in performance which may be modulated by the task-uninformative visual cues. The spatially aligned visual cues may correct a bias in the subject’s model and result in improved performance. In such cases, even the responses of subjects performing exact inference may affected by task-uninformative cues.

**Approximate inference** of posteriors by the subjects may be able to explain the difference in performance seen empirically. The performance of the ideal observer depends only on the side where the posterior over the tone location has higher mass and not the relative magnitude of the posterior on both sides. However, for subjects performing approximate inference (e.g. probability matching), the relative magnitude will influence performance. Since that is modulated by the visual cues, those cues would influence the performance of the subjects. Furthermore, formal model comparison suggests that observers performing simple audio-visual localization may use this strategy [27].

## Conclusion

Here we show that listeners use task-uninformative visual stimuli to improve their performance on an auditory spatial discrimination task. This finding demonstrates that the brain can pair auditory and visual stimuli in a more complex environment than typically created in the lab to improve judgments about relative auditory position. The failing of the ideal Bayesian causal inference model to replicate this effect also indicates that these listeners deviate from ideal observers in systematic ways that may lead to insights into the underlying multisensory mechanisms.

## Methods

### Psychophysics

24 Participants (14 female, 10 male) between ages of 19–27 years (mean of 22 ± 2) gave written informed consent to participate and were paid for time spent in the lab. Each subject had normal or corrected-to-normal vision and normal hearing (thresholds of 20 dB HL or better for octave frequencies between 500 and 8000 Hz). During the experiment subjects were seated in a dark soundproof booth with a viewing distance of 50 cm from a 24 inch BenQ monitor with the center of the screen approximately lined up with their nose. The monitor refreshed at 120 frames per second at a 1920 by 1080 pixel resolution. Protocol was approved by the University of Rochester Research Subjects Review Board.

#### Stimuli

Two auditory stimuli were generated in the frequency domain with energy from 220 to 4000 Hz and a 1/*f* envelope (−3 dB/octave). One was pink noise (“noise’’) and the other was composed of harmonics of 220 Hz (“tone’’). With the exception of one subject who was run with a frozen noise token, the noise was randomly generated for each trial. Data were similar for the subject with the frozen noise token and therefore not excluded. In order to change the location of each sound, they were convolved with HRTFs (head related transfer functions) from the CIPIC library [28]. Because the experimentally determined HRTFs were only recorded at intervals of 5 degrees in the azimuthal plane, we used angles between 0 and 5 degrees that were generated from interpolated HRTFs (see expyfun). Adapting methods from [29], we generated weights for each of two known HRTFs based on distance from the desired HRTF. Then we took the weighted geometric mean of the known HRTF amplitudes and the weighted arithmetic mean of the angles. After convolution, noise and tone were summed and given a 20 ms raised-cosine ramp at the on and offsets. They were presented at 65 dB SPL at a sampling frequency of 24414 Hz from TDT hardware (Tucker Davis Technologies, Alachua, FL). Auditory stimuli had a duration of 300 ms.

The visual stimuli were regular polygons inscribed in a circle with diameter 1.5 degrees. They were randomly assigned four to eight sides for each trial while ensuring that the two shapes were different. The colors of the shapes were specified according to the HSL scheme, and had constant luminance of 0.6, saturation of 1, and per-trial random hue such that the two shapes in the trial had opposite hue. Each shape was presented during alternating frames at 144 frames per second such that both shapes were visible, even in cases where they would overlap (in a manner similar to [30]).

#### Task

During each trial, the tone and noise were presented symmetrically about zero degrees azimuth with visual onset leading auditory by 100 ms. Trials began when subjects gaze fell within a 100 pixel (roughly 2.5 degree) radius of the fixation point (measured by EyeLink 1000 Plus (SR Research, Ontario, Canada)), visual stimuli appeared for 100 ms before the auditory stimuli, and stayed on the screen until the end of the 300 ms auditory stimuli. Subjects were asked to report which side the tone was on by pressing one of two buttons. At the end of the trial we ensured that the subject’s gaze was within a 200 pixel (roughly 5 degree) radius of the fixation point before logging the trial. Before the experiment, subjects were given 10 sample trials and then asked to complete a training session. Their responses to training trials with auditory stimuli at 20 degrees separation were logged until 20 trials had been completed and with enough correct responses that the probability of achieving above-chance performance by random guessing (assuming a binomial distribution) was under 5%. If the training criteria were not satisfied subjects were allowed to re-attempt once. Four subjects were dismissed when they did not pass the second attempt.

There were two conditions tested: a matched condition in which the visual and auditory stimuli were spatially aligned, and a central condition in which the visual stimuli were located at the center of the screen (providing no information about the auditory stimuli and therefore serving as a control). Within these conditions we tested five different auditory separations: 1.25, 2.5, 5, 10, and 20 degrees. For each separation there were 80 trials (40 with the target on the right and 40 with the target on the left) for a total of 800 trials.

Conditions and separations were randomly interleaved such that the conditions could only lag each other by 2 trials. After subjects got a multiple of 3 trials correct in a row, they were given an encouraging message telling them how many consecutive correct responses they had given. After each set of 40 trials, participants were given a self-timed break.

#### Analysis

We performed maximum likelihood fits to the percent correct of the responses at log transformed auditory separations. First we estimated the lapse rate and slope of each subject by doing a preliminary sigmoidal fit on the pooled responses to both conditions. Then using these estimates of lapse rate and slope, we fit responses for both conditions, central (control) and matched, only letting midpoint vary. The lapse rate and slope should be independent of the visual condition. Thresholds were approximated as the separation level at which the fit crossed 75% correct. Using *p* < 0.05 as the criteria for significance, we compared the matched and central percent correct measures with paired *t*-tests. Because thresholds were not normally distributed across subjects, changes thereof were assessed with a sign test.

### Modeling

**Fig 4:**
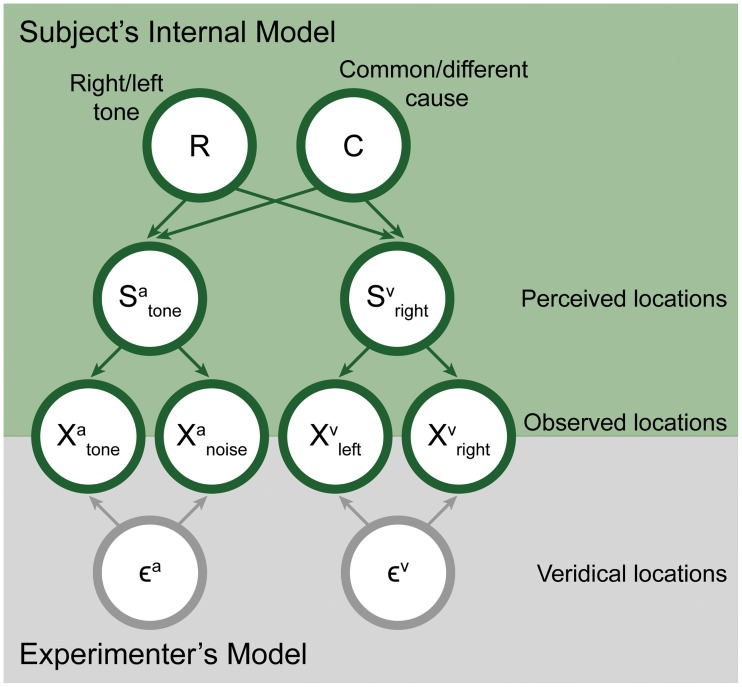
Graphical depiction of our model schematic. Our full model contains two generative models. The first one is the experimenter’s model which maps the true task variables to the sensory observations made by the subject. The second is the subject’s internal model of the sensory observations which is used the subject’s perception (Inference in the generative model).

We model the subject responses from a normative perspective by using an ideal observer model. The subjects are assumed to have learned a generative model of the inputs and base their decision on the inferred tone side. The structure of the model is generally summarized in Fig 4.

#### Model definition for a single trial

For each trial, we denote the true auditory tone location (signed) as $\epsilon_{\text{tone}}^{a}$ and true visual cue eccentricity (always positive) as $\epsilon_{\text{right}}^{v}$ (this is sufficient to define all the inputs since the true noise location and true left visual cue locations are the negatives of the aforementioned values). In this notation, $\text{sign}(\epsilon_{\text{tone}}^{\text{a}})$ denotes the correct response for that trial. Using the notation $N(x;\mu ,\sigma^{2})$ to denote the probability density function of a normal random variable with mean *μ* and variance *σ*^2^, the observed tone location ($X_{\text{tone}}^{a}$), noise location ($X_{\text{noise}}^{a}$), left visual cue location ($X_{\text{left}}^{v}$) and right visual cue location ($X_{\text{right}}^{v}$) for the trial are randomly drawn with probability:

$$\begin{array}{c} P\left(X_{\text{tone}}^{a}|\epsilon_{\text{tone}}^{a}\right)=N\left(X_{\text{tone}}^{a};\epsilon_{\text{tone}}^{a},{\left(\sigma_{\text{tone}}^{a}\right)}^{2}\right) \end{array}$$ (1)

$$\begin{array}{c} P\left(X_{\text{noise}}^{a}|\epsilon_{\text{tone}}^{a}\right)=N\left(X_{\text{noise}}^{a};-\epsilon_{\text{tone}}^{a},{\left(\sigma_{\text{noise}}^{a}\right)}^{2}\right) \end{array}$$ (2)

$$\begin{array}{c} P\left(X_{\text{left}}^{v}|\epsilon_{\text{right}}^{v}\right)=N\left(X_{\text{left}}^{v};-\epsilon_{\text{right}}^{v},{\left(\sigma^{v}\right)}^{2}\right) \end{array}$$ (3)

$$\begin{array}{c} P\left(X_{\text{right}}^{v}|\epsilon_{\text{right}}^{v}\right)=N\left(X_{\text{right}}^{v};\epsilon_{\text{right}}^{v},{\left(\sigma^{v}\right)}^{2}\right) \end{array}$$ (4)

where ${\left(\sigma_{\text{tone}}^{a}\right)}^{2}$, ${\left(\sigma_{\text{noise}}^{a}\right)}^{2}$, ${\left(\sigma^{v}\right)}^{2}$ are the uncertainties associated with the observed tone, noise and visual cue locations respectively.

It is important to note that the subject does not have access to the true variables $\epsilon_{\text{tone}}^{a}$ and $\epsilon_{\text{right}}^{v}$ and must make their decision from the observed variables.

We model subject perception as inference in a hierarchical generative model of the sensory inputs (shown in the figure). Let $S_{\text{tone}}^{a}$ and $S_{\text{right}}^{v}$ be the perceived tone and right visual cue location whose likelihood are given as follows

$$\begin{array}{c} P\left(X_{\text{tone}}^{a}|S_{\text{tone}}^{a}\right)=N\left(X_{\text{tone}}^{a};S_{\text{tone}}^{a},{\left(\sigma_{\text{tone}}^{a}\right)}^{2}\right) \end{array}$$ (5)

$$\begin{array}{c} P\left(X_{\text{noise}}^{a}|S_{\text{tone}}^{a}\right)=N\left(X_{\text{noise}}^{a};-S_{\text{tone}}^{a},{\left(\sigma_{\text{noise}}^{a}\right)}^{2}\right) \end{array}$$ (6)

$$\begin{array}{c} P\left(X_{\text{left}}^{v}|S_{\text{right}}^{v}\right)=N\left(X_{\text{left}}^{v};-S_{\text{right}}^{v},{\left(\sigma^{v}\right)}^{2}\right) \end{array}$$ (7)

$$\begin{array}{c} P\left(X_{\text{right}}^{v}|S_{\text{right}}^{v}\right)=N\left(X_{\text{right}}^{v};S_{\text{right}}^{v},{\left(\sigma^{v}\right)}^{2}\right) \end{array}$$ (8)

Eqs 5 to 8 assume that the subjects can account for their uncertainty accurately based on prior sensory experience. We assume that the subject has learned that the auditory and visual stimuli are symmetric about zero degrees azimuth, which allows them to collapse $X_{\text{tone}}^{a}$ (or $X_{\text{left}}^{v}$) and $X_{\text{noise}}^{a}$ (or $X_{\text{right}}^{v}$) into unimodal estimates. A more general approach would be to assume that the subject performs causal inference to determine if the tone and the noise from the same eccentricity or not. If the subject infers that the tone and noise do not come from the same eccentricity (possible due to sensor noise), they would only consider the likelihood over the tone which would not change the conclusions as the likelihood has the same form, just with higher variance. The priors over $S_{\text{tone}}^{a}$ and $S_{\text{right}}^{v}$ can be conditioned on whether the subject perceived the tone to be from left or right (denoted as R = -1 or R = 1 respectively) and if they perceived the auditory and visual cues to be from the same cause or not (denoted by C = 1 or C = 0 respectively). Assuming a flat prior over location for $S_{\text{tone}}^{a}$ and $S_{\text{right}}^{v}$ (the results still hold for symmetric proper priors), this can be written as

$$\begin{array}{c} P\left(S_{\text{tone}}^{a},S_{\text{right}}^{v}|R,C\right)\propto_{R}\left(\left(1-C\right)+C\delta \left(S_{\text{right}}^{v}-RS_{\text{tone}}^{a}\right)\right)H\left(S_{\text{right}}^{v}\right)H\left(RS_{\text{tone}}^{a}\right) \end{array}$$ (9)

where ∝_*R*_ indicates that the proportionality context is independent of R. *H*(*x*) denotes the Heaviside function.

Having inferred R, we note that the ideal observer makes their choice (Ch) by choosing the side with the higher posterior mass, i.e.

$$\begin{array}{c} P\left(Ch|X_{\text{tone}}^{a},X_{\text{noise}}^{a},X_{\text{left}}^{v},X_{\text{right}}^{v}\right)=\delta \left(Ch-\text{arg}\;\underset{R}{\text{max}}\;P\left(R|X_{\text{tone}}^{a},X_{\text{noise}}^{a},X_{\text{left}}^{v},X_{\text{right}}^{v}\right)\right) \end{array}$$ (10)

#### Calculating the posterior

Before comparing the probability mass on either side, we must evaluate the posterior over R. In order to do so, we marginalize over the cause variable C

$$\begin{array}{c} P\left(R|X_{\text{tone}}^{a},X_{\text{noise}}^{a},X_{\text{left}}^{v},X_{\text{right}}^{v}\right)=\sum_{C\in \{0,1\}}P\left(R,C|X_{\text{tone}}^{a},X_{\text{noise}}^{a},X_{\text{left}}^{v},X_{\text{right}}^{v}\right) \end{array}$$ (11)

We can evaluate the term inside the sum by first using Bayes rule and then simplifying under the assumption that the priors over R and C are assumed to be independent, i.e. *P*(*R*, *C*) = *P*(*R*)*P*(*C*)

$$\begin{array}{c} P\left(R|X_{\text{tone}}^{a},X_{\text{noise}}^{a},X_{\text{left}}^{v},X_{\text{right}}^{v}\right)\propto_{R}\sum_{C\in \{0,1\}}P\left(X_{\text{tone}}^{a},X_{\text{noise}}^{a},X_{\text{left}}^{v},X_{\text{right}}^{v}|R,C\right)P\left(R,C\right) \end{array}$$ (12)

$$\begin{array}{c} P\left(R|X_{\text{tone}}^{a},X_{\text{noise}}^{a},X_{\text{left}}^{v},X_{\text{right}}^{v}\right)\propto_{R}P\left(R\right)\sum_{C\in \{0,1\}}P\left(X_{\text{tone}}^{a},X_{\text{noise}}^{a},X_{\text{left}}^{v},X_{\text{right}}^{v}|R,C\right)P\left(C\right) \end{array}$$ (13)

By assuming equal priors for the left and right side, i.e. *P*(*R*) = 0.5(Ideal observer has no response bias.

$$\begin{array}{c} P\left(R|X_{\text{tone}}^{a},X_{\text{noise}}^{a},X_{\text{left}}^{v},X_{\text{right}}^{v}\right)\propto_{R}\sum_{C\in \{0,1\}}P\left(X_{\text{tone}}^{a},X_{\text{noise}}^{a},X_{\text{left}}^{v},X_{\text{right}}^{v}|R,C\right)P\left(C\right) \end{array}$$ (14)

We can then expand the expression of the side with the higher posterior mass by considering both values of the cause variable C, which using Eq 14 can be rewritten as

$$\begin{array}{c} \text{arg}\;\underset{R}{\text{max}}\;P\left(R|X_{\text{tone}}^{a},X_{\text{noise}}^{a},X_{\text{left}}^{v},X_{\text{right}}^{v}\right)=\\ \;\;\text{arg}\;\underset{R}{\text{max}}\;P\left(X_{\text{tone}}^{a},X_{\text{noise}}^{a},X_{\text{left}}^{v},X_{\text{right}}^{v}|R,C=0\right)P\left(C=0\right)\\ \;\;\;+P\left(X_{\text{tone}}^{a},X_{\text{noise}}^{a},X_{\text{left}}^{v},X_{\text{right}}^{v}|R,C=1\right)P\left(C=1\right) \end{array}$$ (15)

In general, the likelihood can be evaluated by averaging over all possible auditory and visual cue locations

$$\begin{array}{c} P(X_{\text{tone}}^{a},X_{\text{noise}}^{a},X_{\text{left}}^{v},X_{\text{right}}^{v}|R,C)=\\ \int_{-\infty }^{\infty }\int_{-\infty }^{\infty }P\left(X_{\text{tone}}^{a},X_{\text{noise}}^{a},X_{\text{left}}^{v},X_{\text{right}}^{v}|S_{\text{tone}}^{a},S_{\text{right}}^{v}\right)P\left(S_{\text{tone}}^{a},S_{\text{right}}^{v}|R,C\right)dS_{\text{tone}}^{a}dS_{\text{right}}^{v} \end{array}$$ (16)

Using the independence relations implied by the generative model, we can simplify the previous equation to get

$$\begin{array}{c} P(X_{\text{tone}}^{a},X_{\text{noise}}^{a},X_{\text{left}}^{v},X_{\text{right}}^{v}|R,C)=\ldots \\ \int_{-\infty }^{\infty }\int_{-\infty }^{\infty }P\left(X_{\text{tone}}^{a}|S_{\text{tone}}^{a}\right)P\left(X_{\text{noise}}^{a}|S_{\text{tone}}^{a}\right)P\left(X_{\text{left}}^{v}|S_{\text{right}}^{v}\right)P\left(X_{\text{right}}^{v}|S_{\text{right}}^{v}\right)\ldots \\ P\left(S_{\text{tone}}^{a},S_{\text{right}}^{v}|R,C\right)dS_{\text{tone}}^{a}dS_{\text{right}}^{v} \end{array}$$ (17)

Substituting expressions for the likelihoods of each cue (Eqs 5–8) and the prior (Eq 9), we can evaluate Eq 17 by repeated multiplication of normal probability density functions to get expressions for both *C* = 0 and *C* = 1.

#### No audio-visual combination (C = 0)

$$\begin{array}{c} P(X_{\text{tone}}^{a},X_{\text{noise}}^{a},X_{\text{left}}^{v},X_{\text{right}}^{v}|R,C=0)=\ldots \\ \;\int_{-\infty }^{\infty }N\left(X_{\text{tone}}^{a};S_{\text{tone}}^{a},{\left(\sigma_{\text{tone}}^{a}\right)}^{2}\right)N\left(X_{\text{noise}}^{a};-S_{\text{tone}}^{a},{\left(\sigma_{\text{noise}}^{a}\right)}^{2}\right)H\left(RS_{\text{tone}}^{a}\right)dS_{\text{tone}}^{a}\ldots \\ \;\int_{-\infty }^{\infty }N\left(X_{\text{left}}^{v};-S_{\text{right}}^{v},{\left(\sigma^{v}\right)}^{2}\right)N\left(X_{\text{right}}^{v};S_{\text{right}}^{v},{\left(\sigma^{v}\right)}^{2}\right)H\left(S_{\text{right}}^{v}\right)dS_{\text{right}}^{v} \end{array}$$ (18)

Multiplying the gaussian likelihoods in Eq 18, we can pull the terms independent of R into a proportionality constant to get

$$\begin{array}{c} P\left(X_{\text{tone}}^{a},X_{\text{noise}}^{a},X_{\text{left}}^{v},X_{\text{right}}^{v}|R,C=0\right)\propto_{R}\\ \;\int_{-\infty }^{\infty }N\left(X_{\text{tone},\text{noise}}^{a};S_{\text{tone}}^{a},{\left(\sigma_{\text{tone}}^{a}\right)}^{2}\alpha_{\text{tone},\text{noise}}^{a}\right)H\left(RS_{\text{tone}}^{a}\right)dS_{\text{tone}}^{a} \end{array}$$ (19)

where

$$\begin{array}{c} \alpha_{\text{tone},\text{noise}}^{a}=\frac{{\left(\sigma_{\text{noise}}^{a}\right)}^{2}}{{\left(\sigma_{\text{tone}}^{a}\right)}^{2}+{\left(\sigma_{\text{noise}}^{a}\right)}^{2}} \end{array}$$

is the weight given to the tone location while combining with the noise location.

$$\begin{array}{c} X_{\text{tone},\text{noise}}^{a}=X_{\text{tone}}^{a}\alpha_{\text{tone},\text{noise}}^{a}-\left(1-\alpha_{\text{tone},\text{noise}}^{a}\right)X_{\text{noise}}^{a} \end{array}$$

is the combined estimate of the auditory tone location by weighting the tone and noise observation by their inverse variances. The integral in Eq 19 is the area of the combined gaussian likelihood for the tone and noise on either the positive or negative side of 0 depending on R.

$$\begin{array}{c} P\left(X_{\text{tone}}^{a},X_{\text{noise}}^{a},X_{\text{left}}^{v},X_{\text{right}}^{v}|R,C=0\right)\propto_{R}\Phi \left(0;-RX_{\text{tone},\text{noise}}^{a},{\left(\sigma_{\text{tone}}^{a}\right)}^{2}\alpha_{\text{tone},\text{noise}}^{a}\right) \end{array}$$ (20)

where Φ(*x*; *μ*, *σ*^2^) denotes the cumulative density function evaluated at x for a normal random variable with mean *μ* and variance *σ*^2^.

#### Audio-visual cue combination (C = 1)

$$\begin{array}{c} P(X_{\text{tone}}^{a},X_{\text{noise}}^{a},X_{\text{left}}^{v},X_{\text{right}}^{v}|R,C=1)=\ldots \\ \;\int_{-\infty }^{\infty }\int_{-\infty }^{\infty }N\left(X_{\text{tone}}^{a};S_{\text{tone}}^{a},{\left(\sigma_{\text{tone}}^{a}\right)}^{2}\right)N\left(X_{\text{noise}}^{a};-S_{\text{tone}}^{a},{\left(\sigma_{\text{noise}}^{a}\right)}^{2}\right)\ldots \\ \;\;N\left(X_{\text{left}}^{v};-S_{\text{right}}^{v},{\left(\sigma^{v}\right)}^{2}\right)N\left(X_{\text{right}}^{v};S_{\text{right}}^{v},{\left(\sigma^{v}\right)}^{2}\right)\delta \left(S_{\text{right}}^{v}-RS_{\text{tone}}^{a}\right)...\\ \;\;\;\;\;H\left(RS_{\text{tone}}^{a}\right)H\left(S_{\text{right}}^{v}\right)dS_{\text{tone}}^{a}dS_{\text{right}}^{v} \end{array}$$ (21)

We can integrate over $S_{\text{right}}^{v}$ by evaluating all functions of $S_{\text{right}}^{v}$ at $RS_{\text{tone}}^{a}$ because of $\delta (S_{\text{right}}^{v}-RS_{\text{tone}}^{a})$

$$\begin{array}{c} P(X_{\text{tone}}^{a},X_{\text{noise}}^{a},X_{\text{left}}^{v},X_{\text{right}}^{v}|R,C=1)=\ldots \\ \;\int_{-\infty }^{\infty }N\left(X_{\text{tone}}^{a};S_{\text{tone}}^{a},{\left(\sigma_{\text{tone}}^{a}\right)}^{2}\right)N\left(X_{\text{noise}}^{a};-S_{\text{tone}}^{a},{\left(\sigma_{\text{noise}}^{a}\right)}^{2}\right)\ldots \\ \;\;N\left(X_{\text{left}}^{v};-RS_{\text{tone}}^{a},{\left(\sigma^{v}\right)}^{2}\right)N\left(X_{\text{right}}^{v};RS_{\text{tone}}^{a},{\left(\sigma^{v}\right)}^{2}\right)H\left(RS_{\text{tone}}^{a}\right)dS_{\text{tone}}^{a} \end{array}$$ (22)

Multiplying the gaussian likelihoods in Eq 22, we can pull the terms independent of R into a proportionality constant to get

$$\begin{array}{c} P\left(X_{\text{tone}}^{a},X_{\text{noise}}^{a},X_{\text{left}}^{v},X_{\text{right}}^{v}|R,C=0\right)\propto_{R}\\ \;N(RX_{\text{tone},\text{noise}}^{a},\left(\frac{X_{\text{right}}^{v}-X_{\text{left}}^{v}}{2}\right),{\left(\sigma_{\text{tone}}^{a}\right)}^{2}\alpha_{\text{tone},\text{noise}}^{a}+\frac{{\left(\sigma^{v}\right)}^{2}}{2})...\\ \;\;\int_{-\infty }^{\infty }N\left(X_{\text{combined}}^{a,v};S_{\text{tone}}^{a},{\left(\sigma_{\text{tone}}^{a}\right)}^{2}\alpha_{\text{tone},\text{noise}}^{a}\alpha^{av}\right)H\left(RS_{\text{tone}}^{a}\right)dS_{\text{tone}}^{a} \end{array}$$ (23)

where

$$\begin{array}{c} \alpha^{av}=\frac{0.5{\left(\sigma^{v}\right)}^{2}}{{\left(\sigma_{\text{tone}}^{a}\right)}^{2}\alpha_{\text{tone},\text{noise}}^{a}+0.5{\left(\sigma^{v}\right)}^{2}} \end{array}$$

is the weight given to auditory location while combining with the visual location and

$$\begin{array}{c} X_{\text{combined}}^{a,v}=X_{\text{tone},\text{noise}}^{a}\alpha^{av}+R\left(1-\alpha^{av}\right)\left(\frac{X_{\text{right}}^{v}-X_{\text{left}}^{v}}{2}\right) \end{array}$$

is the weighted combination of the visual and auditory cues. While we have combined the information within the same sensory modality and then combined the information across the sensory modalities, the order can be interchanged as it is equivalent to changing the order of multiplication of the four terms in Eq 21. The integral in Eq 23 evaluates to

$$\begin{array}{c} P\left(X_{\text{tone}}^{a},X_{\text{noise}}^{a},X_{\text{left}}^{v},X_{\text{right}}^{v}|R,C=1\right)\propto_{R}\\ \;N(RX_{\text{tone},\text{noise}}^{a},\left(\frac{X_{\text{right}}^{v}-X_{\text{left}}^{v}}{2}\right),{\left(\sigma_{\text{tone}}^{a}\right)}^{2}\alpha_{\text{tone},\text{noise}}^{a}+\frac{{\left(\sigma^{v}\right)}^{2}}{2})...\\ \;\;\Phi (0;-RX_{\text{combined}}^{a,v},{\left(\sigma_{\text{tone}}^{a}\right)}^{2}\alpha_{\text{tone},\text{noise}}^{a}\alpha^{av}) \end{array}$$ (24)

Using the fact that Φ(0; *μ*, *σ*^2^) is a decreasing function of *μ*, the maximum of Eq 20 simplifies to

$$\begin{array}{c} \text{arg}\;\underset{R}{\text{max}}\;P\left(X_{\text{tone}}^{a},X_{\text{noise}}^{a},X_{\text{left}}^{v},X_{\text{right}}^{v}|R,C=0\right)=\text{sign}\left(RX_{\text{tone},\text{noise}}^{a}\right) \end{array}$$ (25)

We note that $(\frac{X_{\text{right}}^{v}-X_{\text{left}}^{v}}{2})>0$ (by definition). Using that fact $N\left(sign\left(\mu \right)x,\mu ,\sigma^{2}\right)>N\left(-sign\left(\mu \right)x,\mu ,\sigma^{2}\right)$ in addition to the decreasing nature of Φ(0; *μ*, *σ*^2^), the maximum of Eq 24 simplifies to

$$\begin{array}{c} \text{arg}\;\underset{R}{\text{max}}\;P\left(X_{\text{tone}}^{a},X_{\text{noise}}^{a},X_{\text{left}}^{v},X_{\text{right}}^{v}|R,C=1\right)\\ \;\;\;=\text{sign}(RX_{\text{tone},\text{noise}}^{a})\\ \;\;\;=\text{arg}\;\underset{R}{\text{max}}\;P\left(X_{\text{tone}}^{a},X_{\text{noise}}^{a},X_{\text{left}}^{v},X_{\text{right}}^{v}|R,C=0\right) \end{array}$$ (26)

The positive weighted combination of two function is maximized at the point of maximization of the individual functions if the individual point of maximizations are equal. Using this result, we can substitute Eq 21 into Eq 15 to get

$$\begin{array}{c} \text{arg}\;\underset{R}{\text{max}}\;P\left(R|X_{\text{tone}}^{a},X_{\text{noise}}^{a},X_{\text{left}}^{v},X_{\text{right}}^{v}\right)=\text{sign}\left(RX_{\text{tone},\text{noise}}^{a}\right) \end{array}$$ (27)

Importantly, the side with the higher posterior mass is independent of cause C.

#### Generating a psychometric curve

To evaluate the probability the subject will choose right at each auditory azimuth (psychometric curve), we need

$$\begin{array}{c} P(Ch=1|\epsilon_{\text{tone}}^{a},\epsilon_{\text{right}}^{v})=\\ \;\;\int_{-\infty }^{\infty }\int_{-\infty }^{\infty }\int_{-\infty }^{\infty }\int_{-\infty }^{\infty }P\left(Ch=1|X_{\text{tone}}^{a},X_{\text{noise}}^{a},X_{\text{left}}^{v},X_{\text{right}}^{v}\right)\\ \;\;P\left(X_{\text{tone}}^{a},X_{\text{noise}}^{a},X_{\text{left}}^{v},X_{\text{right}}^{v}|\epsilon_{\text{tone}}^{a},\epsilon_{\text{right}}^{v}\right)dX_{\text{tone}}^{a}dX_{\text{noise}}^{a}dX_{\text{left}}^{v}dX_{\text{right}}^{v} \end{array}$$ (28)

Using the independence relations implied by the generative model, we can simplify the previous equation to get

$$\begin{array}{c} P(Ch=1|\epsilon_{\text{tone}}^{a},\epsilon_{\text{right}}^{v})=\\ \;\int_{-\infty }^{\infty }\int_{-\infty }^{\infty }\int_{-\infty }^{\infty }\int_{-\infty }^{\infty }P\left(Ch=1|X_{\text{tone}}^{a},X_{\text{noise}}^{a},X_{\text{left}}^{v},X_{\text{right}}^{v}\right)\\ \;P\left(X_{\text{tone}}^{a}|\epsilon_{\text{tone}}^{a}\right)P\left(X_{\text{noise}}^{a}|\epsilon_{\text{tone}}^{a}\right)P\left(X_{\text{left}}^{v}|\epsilon_{\text{right}}^{v}\right)P\left(X_{\text{right}}^{v}|\epsilon_{\text{right}}^{v}\right)\\ dX_{\text{tone}}^{a}dX_{\text{noise}}^{a}dX_{\text{left}}^{v}dX_{\text{right}}^{v} \end{array}$$ (29)

Substituting Eqs 1–4, 27 in Eq 29 and simplifying, we get

$$\begin{array}{c} P\left(Ch=1|\epsilon_{\text{tone}}^{a},\epsilon_{\text{right}}^{v}\right)=\Phi \left(0;-\epsilon_{\text{tone}}^{a},{\left(\sigma_{\text{noise}}^{a}\right)}^{2}\alpha_{\text{tone},\text{noise}}^{a}\right) \end{array}$$ (30)

Assuming a subject lapses with a probability λ and responds randomly with equal probability, we get the model predicted psychometric curve as

$$\begin{array}{c} P\left(Ch=1|\epsilon_{\text{tone}}^{a},\epsilon_{\text{right}}^{v}\right)=\lambda \left(0.5\right)+\left(1-\lambda \right)\Phi \left(0;-\epsilon_{\text{tone}}^{a},{\left(\sigma_{\text{noise}}^{a}\right)}^{2}\alpha_{\text{tone},\text{noise}}^{a}\right) \end{array}$$ (31)

It is important to note that the ideal observer response is **not affected** by the observations of the visual cue location.

### Model fitting

The model described in the previous section has two free parameters to model the subject responses:

- Effective auditory uncertainty [${\left(\sigma_{eff}^{a}\right)}^{2}$]
- Lapse rate (λ)

Because the ideal observer is not affected by visual cues, we do not fit a parameter for visual uncertainty. We compute the posterior over these parameters (denoted as *θ*) from subject responses. Let *n*_condition_ denote the number of stimulus conditions in the experiment and *n*_trial_ denote the number of trials for each condition. We denote the true auditory eccentricity and true right visual cue eccentricity for condition *i* as ${\left(\epsilon_{\text{tone}}^{a}\right)}_{\left(i\right)}$ and ${\left(\epsilon_{\text{right}}^{v}\right)}_{\left(i\right)}$ respectively. The experimental subject responses for these conditions are denoted by (*r*)_(*i*)_ which is modeled as a binomial random variable

$$\begin{array}{c} P\left[{\left(r\right)}_{\left(i\right)}|{\left(\epsilon_{\text{tone}}^{a}\right)}_{\left(i\right)},{\left(\epsilon_{\text{right}}^{v}\right)}_{\left(i\right)},\theta \right]=Bin\left\{n_{\text{trial}},P\left[Ch=1|{\left(\epsilon_{\text{tone}}^{a}\right)}_{\left(i\right)},{\left(\epsilon_{\text{right}}^{v}\right)}_{\left(i\right)},\theta \right]\right\} \end{array}$$ (32)

where *Bin*(*n*, *p*) denotes the binomial probability density function with parameters *n* and *p*. The probability parameter in Eq 32 is obtained from Eq 31 for the parameter values.

Given these data points from the experiment, we are interested in calculating the probability of the parameter value given this data, i.e. $P(\theta |{\left(\epsilon_{\text{tone}}^{a}\right)}_{1,2,..n_{\text{condition}}},{\left(\epsilon_{\text{right}}^{v}\right)}_{1,2,..n_{\text{condition}}},{\left(r\right)}_{1,2,..n_{\text{condition}}})$. Using Bayes rule,

$$\begin{array}{c} P\left[\theta |{\left(\epsilon_{\text{tone}}^{a}\right)}_{1,2,..n_{\text{condition}}},{\left(\epsilon_{\text{right}}^{v}\right)}_{1,2,..n_{\text{condition}}},{\left(r\right)}_{1,2,..n_{\text{condition}}}\right]\propto_{\theta }\\ \;\;\;P\left[{\left(r\right)}_{1,2,..n_{\text{condition}}}|{\left(\epsilon_{\text{tone}}^{a}\right)}_{1,2,..n_{\text{condition}}},{\left(\epsilon_{\text{right}}^{v}\right)}_{1,2,..n_{\text{condition}}},\theta \right]P\left(\theta \right) \end{array}$$ (33)

We have assumed that the probability of the subject’s parameters are independent of the cue location. Assuming all conditions are independent given the parameter value (which is assumed to have a flat prior), we simplify Eq 33 to get

$$\begin{array}{c} \;P\left[\theta |{\left(\epsilon_{\text{tone}}^{a}\right)}_{1,2,..n_{\text{condition}}},{\left(\epsilon_{\text{right}}^{v}\right)}_{1,2,..n_{\text{condition}}},{\left(r\right)}_{1,2,..n_{\text{condition}}}\right]\propto_{\theta }\\ \prod_{i=1}^{n_{\text{condition}}}P\left[{\left(r\right)}_{\left(i\right)}|{\left(\epsilon_{\text{tone}}^{a}\right)}_{\left(i\right)},{\left(\epsilon_{\text{right}}^{v}\right)}_{\left(i\right)},\theta \right] \end{array}$$ (34)

where the term inside the product is given in Eq 32.

We can find the parameters that best fit the data (denoted as θ*) by finding the maximum a posteriori (MAP) solution for Eq (Also the maximum likelihood since the prior is flat). This is often implemented as minimizing the negative log posterior (since log is monotonic)

$$\begin{array}{c} \theta^{*}=\text{arg}\;\underset{\theta }{\text{min}}\sum_{i}^{n_{\text{condition}}}-log\left\{Bin\left\{n_{\text{trial}},P\left[Ch=1|{\left(\epsilon_{\text{tone}}^{a}\right)}_{\left(i\right)},{\left(\epsilon_{\text{right}}^{v}\right)}_{\left(i\right)},\theta \right]\right\}\right\} \end{array}$$ (35)

We optimized Eq 35 using Bayesian adaptive direct search (BADS) [31]. BADS alternates between a series of fast, local Bayesian optimization steps and a systematic, slower exploration of a mesh grid.

## References

[1] Ernst MO, Banks MS. Humans integrate visual and haptic information in a statistically optimal fashion. Nature. 2002;415(6870):429–433. 10.1038/415429a 11807554

[2] Körding KP, Beierholm U, Ma WJ, Quartz S, Tenenbaum JB, Shams L. Causal Inference in Multisensory Perception. PLOS ONE. 2007;2(9):e943 10.1371/journal.pone.0000943 17895984 PMC1978520

[3] Battaglia PW, Jacobs RA, Aslin RN. Bayesian integration of visual and auditory signals for spatial localization. JOSA A. 2003;20(7):1391–1397. 10.1364/JOSAA.20.001391 12868643

[4] Alais D, Burr D. The Ventriloquist Effect Results from Near-Optimal Bimodal Integration. Current Biology. 2004;14(3):257–262. 10.1016/j.cub.2004.01.029 14761661

[5] Shams L, Ma WJ, Beierholm U. Sound-induced flash illusion as an optimal percept. NeuroReport. 2005;16(17):1923 10.1097/01.wnr.0000187634.68504.bb 16272880

[6] Bresciani JP, Dammeier F, Ernst MO. Vision and touch are automatically integrated for the perception of sequences of events. Journal of Vision. 2006;6(5):2–2. 10.1167/6.5.2 16881788

[7] Wozny DR, Beierholm UR, Shams L. Human trimodal perception follows optimal statistical inference. Journal of Vision. 2008;8(3):24–24. 10.1167/8.3.24 18484830

[8] Beierholm UR, Quartz SR, Shams L. Bayesian priors are encoded independently from likelihoods in human multisensory perception. Journal of Vision. 2009;9(5):23–23. 10.1167/9.5.23 19757901

[9] Hospedales T, Vijayakumar S. Multisensory Oddity Detection as Bayesian Inference. PLOS ONE. 2009;4(1):e4205 10.1371/journal.pone.0004205 19145254 PMC2625446

[10] Girshick AR, Banks MS. Probabilistic combination of slant information: Weighted averaging and robustness as optimal percepts. Journal of vision. 2009;9(9):8.1–820. 10.1167/9.9.8 PMC2940417 19761341

[11] Rohe T, Noppeney U. Cortical Hierarchies Perform Bayesian Causal Inference in Multisensory Perception. PLOS Biology. 2015;13(2):e1002073 10.1371/journal.pbio.1002073 25710328 PMC4339735

[12] Rohe T, Noppeney U. Distinct Computational Principles Govern Multisensory Integration in Primary Sensory and Association Cortices. Current Biology. 2016;26(4):509–514. 10.1016/j.cub.2015.12.056 26853368

[13] Maddox RK, Atilgan H, Bizley JK, Lee AK. Auditory selective attention is enhanced by a task-irrelevant temporally coherent visual stimulus in human listeners. eLife. 2015;4 10.7554/eLife.04995 25654748 PMC4337603

[14] Atilgan H, Town SM, Wood KC, Jones GP, Maddox RK, Lee AKC, et al Integration of Visual Information in Auditory Cortex Promotes Auditory Scene Analysis through Multisensory Binding. Neuron. 2018;97(3):640–655.e4. 10.1016/j.neuron.2017.12.034 29395914 PMC5814679

[15] Eramudugolla R, Kamke MR, Soto-Faraco S, Mattingley JB. Perceptual load influences auditory space perception in the ventriloquist aftereffect. Cognition. 2011;118(1):62–74. 10.1016/j.cognition.2010.09.009 20979992

[16] Howard IP, Templeton WB. Human spatial orientation. Human spatial orientation. Oxford, England: John Wiley & Sons; 1966.

[17] Slutsky DA, Recanzone GH. Temporal and spatial dependency of the ventriloquism effect. NeuroReport. 2001;12(1):7 10.1097/00001756-200101220-00009 11201094

[18] Blau V, Atteveldt NV, Formisano E, Goebel R, Blomert L. Task-irrelevant visual letters interact with the processing of speech sounds in heteromodal and unimodal cortex. European Journal of Neuroscience. 2008;28(3):500–509. 10.1111/j.1460-9568.2008.06350.x 18702722

[19] Lovelace CT, Stein BE, Wallace MT. An irrelevant light enhances auditory detection in humans: a psychophysical analysis of multisensory integration in stimulus detection. Cognitive Brain Research. 2003;17(2):447–453. 10.1016/S0926-6410(03)00160-5 12880914

[20] Soto-Faraco S, Navarra J, Alsius A. Assessing automaticity in audiovisual speech integration: evidence from the speeded classification task. Cognition. 2004;92(3):B13–B23. 10.1016/j.cognition.2003.10.005 15019556

[21] Larson E, Lee AKC. The cortical dynamics underlying effective switching of auditory spatial attention. NeuroImage. 2013;64:365–370. 10.1016/j.neuroimage.2012.09.006 22974974 PMC3508251

[22] Best V, Gallun FJ, Ihlefeld A, Shinn-Cunningham BG. The influence of spatial separation on divided listening. The Journal of the Acoustical Society of America. 2006;120(3):1506–1516. 10.1121/1.2234849 17004472

[23] Gallun FJ, Mason CR, Kidd G. Task-dependent costs in processing two simultaneous auditory stimuli. Perception & Psychophysics. 2007;69(5):757–771. 10.3758/BF03193777 17929698

[24] Talsma D, Doty TJ, Woldorff MG. Selective Attention and Audiovisual Integration: Is Attending to Both Modalities a Prerequisite for Early Integration? Cerebral Cortex. 2007;17(3):679–690. 10.1093/cercor/bhk016 16707740

[25] Bizley JK, Maddox RK, Lee AKC. Defining Auditory-Visual Objects: Behavioral Tests and Physiological Mechanisms. Trends in Neurosciences. 2016;39(2):74–85. 10.1016/j.tins.2015.12.007 26775728 PMC4738154

[26] Bizley JK, Jones GP, Town SM. Where are multisensory signals combined for perceptual decision-making? Current Opinion in Neurobiology. 2016;40:31–37. 10.1016/j.conb.2016.06.003 27344253

[27] Wozny DR, Beierholm UR, Shams L. Probability Matching as a Computational Strategy Used in Perception. PLOS Computational Biology. 2010;6(8):e1000871 10.1371/journal.pcbi.1000871 20700493 PMC2916852

[28] Algazi VR, Duda RO, Thompson DM, Avendano C. The CIPIC HRTF database. In: Proceedings of the 2001 IEEE Workshop on the Applications of Signal Processing to Audio and Acoustics (Cat. No.01TH8575). New Platz, NY, USA: IEEE; 2001. p. 99–102. Available from: http://ieeexplore.ieee.org/document/969552/.

[29] Martin R, McAnally K. Interpolation of head-related transfer functions. Defence Science and Technology Organization Ednburgh (Australia) Air Operations Div; 2007.

[30] Blaser E, Pylyshyn ZW, Holcombe AO. Tracking an object through feature space. Nature. 2000;408(6809):196–. 10.1038/35041567 11089972

[31] Acerbi L, Ma WJ. Practical Bayesian Optimization for Model Fitting with Bayesian Adaptive Direct Search. In: Advances in Neural Information Processing Systems; 2017. p. 1836–1846.

